# Mortality and causes of death after surgery for chronic subdural hematoma: a post hoc study of the FINISH randomized trial

**DOI:** 10.1007/s00701-025-06728-9

**Published:** 2025-12-01

**Authors:** Pihla Tommiska, Oula Knuutinen, Kimmo Lönnrot, Teemu Luoto, Ville Leinonen, Timo Koivisto, Sami Tetri, Jussi P. Posti, Rahul Raj

**Affiliations:** 1 Department of Neurosurgery, Helsinki University Hospital and University of Helsinki, Haartmaninkatu 4, Po Box 320, 00029 HUS Helsinki, Finland; 2 Department of Neurosurgery, Oulu University Hospital and University of Oulu, Oulu, Finland; 3 Department of Neurosurgery, Tampere University Hospital and Tampere University, Tampere, Finland; 4 Department of Neurosurgery, Kuopio University Hospital and Institute of Clinical Medicine, University of Eastern Finland, Kuopio, Finland; 5 Department of Neurosurgery and Turku Brain Injury Center, Neurocenter, Turku University Hospital, Turku, Finland; 6 Division of Clinical Neurosciences, Neurosurgery, University of Turku, Turku, Finland

**Keywords:** Chronic subdural hematoma, Mortality, Cause of death, Comorbidity, Neurosurgery

## Abstract

**Purpose:**

Chronic subdural hematoma (CSDH) is a common neurosurgical disease, especially prevalent among the elderly and is associated with reduced life expectancy. This study investigated mortality and causes of death after burr-hole drainage surgery for CSDH.

**Methods:**

We included patients from the FINISH trial, a national, multicenter, randomized study conducted in Finland during 2020–2022. We obtained mortality data from Statistics Finland. For the classification of causes of death, we used the European shortlist of 86 causes, which is derived from the 10th revision of the International Classification of Diseases and Related Health Problems (ICD-10).

**Results:**

Overall, the FINISH trial population included 589 patients (median age 78 years, 28% women). After a median follow-up of 16.4 months (IQR 9.7–23.1), 82 patients (14%) died. The median age at death was 85 years (IQR 81–89), and the median time from surgery to death was 6.5 months (IQR 2.4–15.3). The leading causes of death were circulatory diseases (34%), accidents (16%), and dementia (15%). A higher number of pre-existing comorbidities was significantly associated with increased mortality. In particular, dementia, cardiac arrhythmia, prior cerebrovascular events, and hypertension emerged as significant risk factors for death.

**Conclusion:**

This study provides valuable insights into mortality rates and causes of death among patients undergoing CSDH surgery. The findings underscore the critical role of pre-existing comorbidities in influencing patient outcomes.

**Trial Registration:**

The FINISH trial was registered with ClinicalTrials.gov (NCT04203550) on Dec 16, 2019. The trial is completed.

**Supplementary Information:**

The online version contains supplementary material available at 10.1007/s00701-025-06728-9.

## Introduction

Chronicsubdural hematoma (CSDH) is a collection of blood and its degradation products resulting from head trauma and subsequent inflammatory reaction [4]. The formation of CSDH is facilitated by brain atrophy in the elderly [15], who have a higher incidence of this condition [12]. In parallel with aging populations, the incidence of CSDH is rising [12]. Consequently, the global disease burden of CSDH and its impact on healthcare are increasing.

The typical CSDH patient is an elderly individual [7] with comorbidities [12] and a limited life expectancy [13]. CSDH shortens life expectancy in all exposed age groups, with the highest fatality in older adults with frailty and comorbidities [10, 13, 14]. In high-income countries, one-year mortality rates following a diagnosis of CSDH range between 13% [13] and 30% [3, 8]. In addition to short-term mortality, excess mortality has been observed up to 20 years after diagnosis [13]. Yet, few studies have examined specific causes of death in CSDH patients [1, 7, 13].

This study aimed to evaluate the mortality rate and causes of death in patients undergoing burr-hole drainage for CSDH. We performed a post hoc analysis of the FINISH trial (*The Finnish Study of Intraoperative Irrigation Versus Drain Alone After Evacuation of Chronic Subdural Hematoma*), a large national multicenter randomized trial [11].

## Methods

We used data from the FINISH trial, a randomized controlled trial conducted between Jan 1, 2020 and Aug 17, 2022 in Finland [11]. Adult patients with first-ever symptomatic CSDH requiring surgery were randomized to undergo either burr-hole drainage with subdural irrigation or drainage without irrigation. Exclusion criteria included need for surgical procedures other than burr-hole drainage, prior intracranial surgery, presence of a cerebrospinal fluid shunt, coma, inability to cooperate with postoperative subdural drain use, acute infection, and high risk of thrombosis.

In Finland, an underlying cause of death is reported for each deceased person and defined as the disease that initiated the sequence of illnesses leading directly to death [9]. Death certificates are issued by treating physicians or forensic pathologists, reviewed for accuracy by the National Institute for Health and Welfare and Statistics Finland, and finally confirmed by the Ministry of Social Affairs and Health [9]. All death dates and causes of death categorized using the International Classification of Diseases 10th version (ICD-10) codes are reported to Statistics Finland. We obtained mortality data including date and cause of death from Statistics Finland, and further categorized the causes of death according to the 86 causes in the European shortlist [5]. The patient follow-up extended until February 14, 2023 (6 months after the index surgery), or death. The study was approved by the Helsinki University Hospital ethics committee. A written informed consent was received from the patients or their next-of-kin.

We report medians with interquartile ranges (IQRs) for continuous data and number of observations with percentages for categorical variables. We used the χ^2^ test to compare categorical variables between groups and Kaplan–Meier curves to illustrate survival. We used Cox proportional hazards model to estimate hazard ratios (HRs) with 95% confidence intervals (CIs) for mortality. A two-tailed *P* < 0.05 was considered statistically significant. The statistical analysis was done in Stata, version 18 (StataCorp).

## Results

We included 589 patients from the FINISH trial in this study. The most frequent reasons for exclusion were previous intracranial surgery, patients not approached for recruitment, lack or withdrawal of consent, and severely lowered co-operation and clinical suspicion of postoperative subdural drainage failure. The median age at the time of surgery was 78 years (IQR 72–84 years), and 165 patients (28.0%) were women. The median number of predefined comorbidities per patient was one (IQR 1–2), and 128 patients (21.7%) had none of the predefined comorbidities prior to CSDH surgery (eTable 1).

The most common predefined comorbidities were hypertension (57.7%), diabetes (21.7%), and ischemic heart disease or peripheral artery disease (17.0%, eTable 1). Dementia had been previously diagnosed in 71 patients (12.1%). In total, 187 (31.8%) had one comorbidity, 163 (27.7%) had two, 79 (13.4%) had three, and 26 (4.4%) had four predefined comorbidities.

### Mortality

**Fig. 1:**
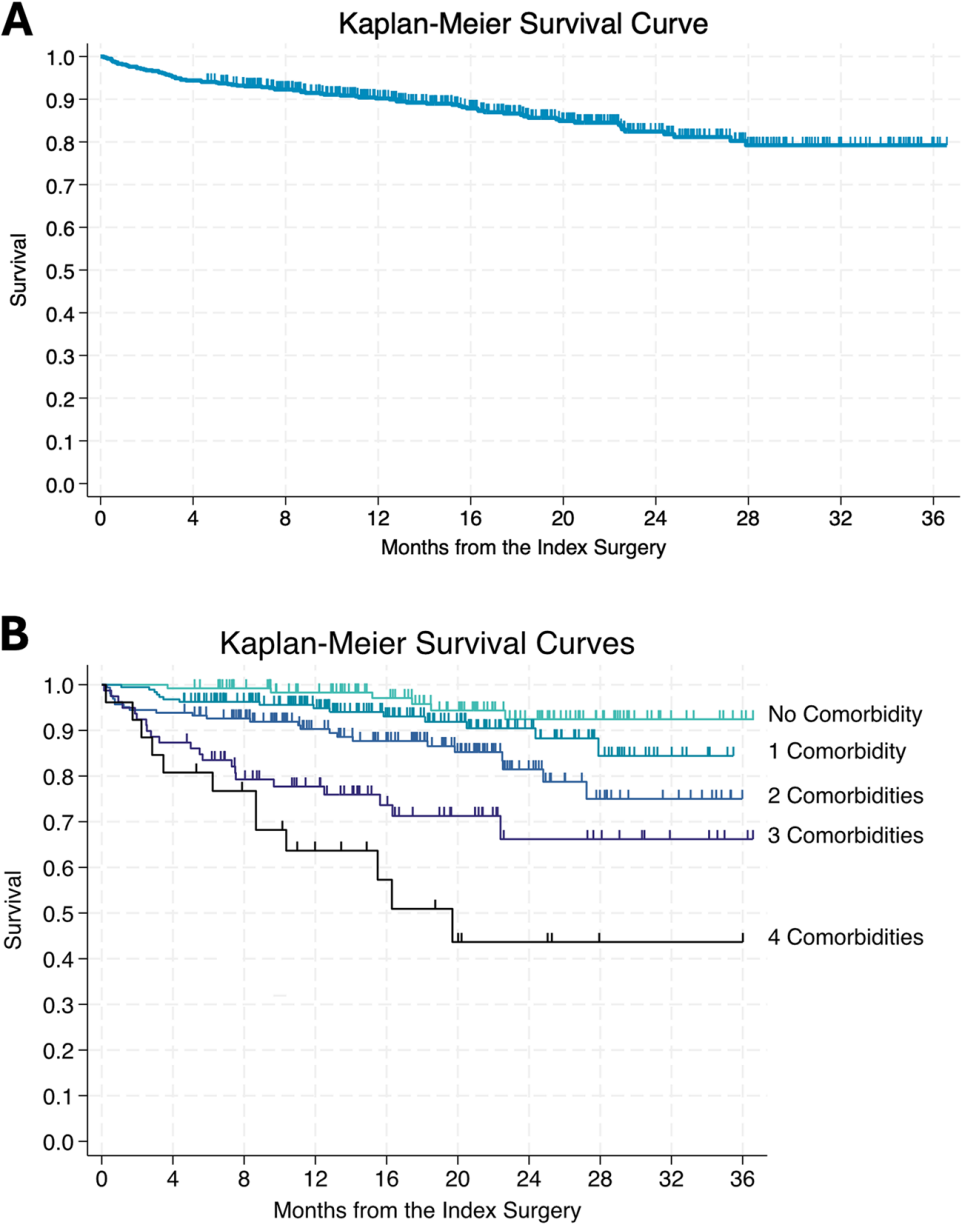
**A** Kaplan–Meier survival curve after chronic subdural hematoma surgery. **B** Kaplan–Meier survival curve after chronic subdural hematoma surgery stratified by preoperative comorbidity counts

During the median follow-up of 16.4 months (IQR 9.7–23.1), 82 of 589 patients (13.9%) died, whereas 6-month mortality was 6.6%. The median age at death was 85 years (IQR 81–89), and the median time from surgery to death was 6.5 months (IQR 2.4–15.3, Fig. 1A).

The number of comorbidities was associated with increased mortality (Fig. 1B). The mortality rates during the complete follow-up were 4.7% for patients with no comorbidities, and increased progressively with the number of comorbidities: 8.0% for one comorbidity, 14.8% for two, 26.6% for three, and 46.2% for four comorbidities (*p* = 0.001). Among specific comorbidities, dementia (HR for death 5.31, 95% CI 3.40–8.30, *p* < 0.001), cardiac arrhythmia (HR 3.23, 95% CI 2.09–4.99, *p* < 0.001), previous cerebrovascular event (HR 2.38, 95% CI 1.46–3.88, *p* = 0.001), and hypertension (HR 1.97, 95% CI 1.22–3.19, *p* = 0.006) were associated with an increased risk for mortality (eFigure 1).

### Causes of death

The most common causes of death were diseases of the circulatory system (*n* = 28/82, 34%), accidents (*n* = 13/82, 16%), and dementia (*n* = 12/82, 15%, Table 1 and eFigure 2A). Diseases of the circulatory system included deaths due to atherosclerotic cardiovascular disease, congestive heart failure, ST-elevation myocardial infarction, mitral valve insufficiency, aneurysmal subarachnoid hemorrhage, intracerebral hemorrhage, embolic cerebral infarction, and vascular cognitive impairment. Accidents included deaths due to traumatic subdural hematoma and sequelae of intracranial injury. Dementia included deaths due to Alzheimer’s disease, vascular dementia, and unspecified dementia.

**Table 1:** Causes of death after surgery for chronic subdural hematoma

Causes of death	Frequency (%)	Age at the time of surgery, median (IQR), years	Time to death from surgery, median (IQR), months	Age at death, median (IQR), years
Diseases of the circulatory system excl. alcohol-related^a^	28 (34%)	86 (82–89)	7 (1–19)	87 (82–90)
Accidents excl. accidental poisoning by alcohol^b^	13 (16%)	89 (81–90)	2 (0–6)	89 (81–91)
Dementia^c^	12 (15%)	87 (83–91)	4 (2–6)	88 (84–91)
Malignant neoplasms	7 (9%)	80 (78–83)	15 (8–17)	81 (80–83)
Other diseases of the nervous system and sense organs excl. alcohol-related^d^	7 (9%)	83 (75–87)	4 (2–14)	83 (75–86)
COVID-19 virus infection	6 (7%)	86 (81–90)	14 (9–19)	88 (82–91)
Alcohol-related diseases and accidental poisoning by alcohol	2 (2%)	55 (43–68)	11 (9–12)	56 (44–69)
Diseases of the digestive system excl. alcohol-related diseases	2 (2%)	84 (83–85)	3 (2–4)	84 (83–85)
Diseases of the genitourinary system	1 (1%)	83	18	84
Diabetes mellitus	1 (1%)	80	16	81
Other diseases excl. alcohol-related	1 (1%)	67	22	69
No death certificate	1 (1%)	94	0	94
Ill-defined and unknown causes of mortality	1 (1%)	76	2	77
All deaths	**82 (100%)**	**78 (72–85)**	**6 (2–15)**	**85 (81–89)**

^a^ Diseases of the circulatory system excluding alcohol-related, included deaths due to atherosclerotic cardiovascular disease, congestive heart failure, ST-elevation myocardial infarction, mitral valve insufficiency, aneurysmal subarachnoid hemorrhage, intracerebral hemorrhage, embolic cerebral infarction, and vascular cognitive impairment

^b^ Accidents excluding accidental poisoning by alcohol included deaths due to traumatic subdural hematoma and sequelae of intracranial injury

^c^ Dementia included deaths due to Alzheimer’s disease, vascular dementia, and unspecified dementia

^d^ Other diseases of the nervous system and sense organs excluding alcohol-related, included deaths due to Parkinson’s disease and motor neuron disease

The median ages at death were 87 years (IQR 82–90 years) for deaths caused by diseases of the circulatory system, 89 years (IQR 81–91 years) for accidents, and 88 years (IQR 84–91 years) for dementia. The median time between death and surgery was seven months (IQR 1–19 months) for deaths caused by diseases of the circulatory system, two months (IQR 0–6) for accidents, and four months (IQR 2–6) for dementia (eFigure 2B). Among accidental causes of death, 12 deaths (*n* = 12/82, 15%) were classified under the ICD-10 code for traumatic subdural hematoma (S06.5), which includes chronic, subacute, and acute subdural hematomas. The median time from surgery to death for these 12 cases was 46 days.

## Discussion

In this post hoc study of the FINISH trial, the 6-month mortality after CSDH surgery was 6.6%, rising to 13.9% over a median follow-up of 16.4 months. The most common causes of death were circulatory diseases, accidents, and dementia. Most patients had at least one comorbidity, and mortality risk increased with comorbidity burden.

The observed 6-month mortality aligns with previous reports [6, 13]. Higher rates (1-year mortality of 20%–32%) have been reported in studies including conservatively treated patients [3, 7, 8]. While some studies suggest excess mortality after CSDH [3, 7, 8, 10, 13], others report rates comparable to the general population [6]. Differences in healthcare systems, life expectancies, and case identification may explain these variations.

We identified three studies reporting specific causes of death among patients with CSDH, and the findings were consistent with our results [1, 7, 13]. The leading causes of death included infections [1, 7], cardiovascular disease [1, 7, 13], dementia [1, 13], neoplasms [1, 13], and accidents [1, 13]. Compared to the general population, CSDH patients had higher mortality from cardiovascular disease [1], accidents [1], dementia [13], and infections [7].

In Finland during 2020–2022, leading causes of death among those aged over 65 were circulatory diseases (34%), dementia (22%), malignant neoplasms (21%), diseases of the nervous system and sense organs (4%), COVID-19 infection (4%), and accidents (4%) [9]. Thus, accidents were more frequent (16% vs. 4%), and malignant neoplasms were less common (9% vs. 21%) causes of death among CSDH patients compared to the general elderly population.

Most patients had at least one pre-existing comorbidity, with only 21.7% having none. Similarly, a large population-based study found that 82% of CSDH patients had at least one comorbidity [13]. As expected, they reported a lower mortality rate among patients without pre-existing comorbidities, consistent with our observations. Prior studies associate ischemic heart disease and dementia with decreased long-term survival after CSDH [7]. These findings along with our results, suggest that the high mortality rates among CSDH patients are likely attributable to their underlying comorbidities.

A strength of this study is its foundation in the FINISH trial, a large, nationwide RCT. The availability and comprehensiveness of data is notably high within the Finnish healthcare system. Furthermore, Finland’s standardized death certification ensures high accuracy and completeness in cause-of-death reporting [9].

This study has several limitations. It is a post hoc analysis of an RCT originally designed to investigate intraoperative irrigation rather than mortality. Preoperative comorbidity data were limited to predefined conditions excluding frailty and other potential confounders such as malignancies, immunosuppressive conditions, neurological disorders, and alcoholism. These unmeasured factors may have influenced mortality and causes of death following CSDH surgery.

Additionally, cause-of-death classification relied solely on ICD-10 codes, preventing us from distinguishing whether deaths attributed to traumatic subdural hematoma resulted from the original CSDH or a new trauma. The median time from surgery to death among the 12 cases classified under the ICD-10 code for traumatic subdural hematoma (S06.5) was 46 days, suggesting that many of these deaths were related to the original CSDH. However, this cannot be confirmed in the present study, as the necessary information was not available. The trial also coincided with the COVID-19 pandemic, with 7% of deaths attributed to COVID-19. Lack of vaccinations [2] early in the study period may have contributed to mortality.

Mortality after CSDH surgery is high and excess mortality has been reported [7, 8, 10, 13]. However, the mortality seems more related to the comorbidities than the hematoma itself. Thus, to reduce mortality, we stress the importance of treating the underlying comorbidities of these frail CSDH patients, as they often die from other diseases than the CSDH. Close collaboration with specialists such as geriatricians and physiotherapists would be highly beneficial in managing these patients. Prospective studies investigating preoperative comorbidities, frailty, and their association with mortality and causes of death following CSDH surgery are needed to enhance patient care and identify those at the highest risk of mortality.

## Conclusion

Our study highlights that patients undergoing CSDH surgery face a high risk of mortality, with diseases of the circulatory system, accidents, and dementia being the leading causes of death. The frequent presence of pre-existing comorbidities increased the risk of mortality. These findings emphasize the necessity of comprehensive care strategies that address both the CSDH and the patients’ underlying comorbidities to improve outcomes.

## Supplementary Information

Below is the link to the electronic supplementary material.

ESM 1 Supplementary Material 1 (DOCX 576 KB)

## Data Availability

All data requests should be submitted to RR by e-mail for consideration. Access to anonymized data may be granted following review and consideration of the Act on the Secondary Use of Health and Social Data in Finland.
