# Supplementary material for: Mortality and causes of death after surgery for chronic subdural hematoma: a post hoc study of the FINISH randomized trial
Source: Acta Neurochir (Wien). 2025 Dec 1;167(1):310. doi: 10.1007/s00701-025-06728-9 (PMC12672672; doi:10.1007/s00701-025-06728-9)
Supplement: Supplementary file 1 — Supplementary Material 1 (DOCX 576 KB) [file 701_2025_6728_MOESM1_ESM.docx]

# Supplemental Material

## Table of Content

**eTable 1.** Patient demographics

**eFigure 1.** Survival by preoperative comorbidity

**eFigure 2A.** Number of deaths for different causes of death

**eFigure 2B.** Time to death from surgery for different causes of death

**Complete list of FINISH investigators**

## Supplementary Tables

eTable 1. Patient demographics

| **Variable** | **Frequency, % (N = 589)** |
| --- | --- |
| Age, median (IQR), years | 78 (72–84) |
| Female sex | 165 (28.0%) |
| Medical comorbidities^a^ |  |
| Diabetes mellitus | 128 (21.7%) |
| Previous cerebrovascular event | 84 (14.3%) |
| Hypertension | 340 (57.7%) |
| Ischemic heart disease or peripheral artery disease | 100 (17.0%) |
| Cardiac valve prosthesis | 2 (0.3%) |
| Pulmonary embolism or deep vein thrombosis^b^ | 13 (2.2%) |
| Dementia | 71 (12.1%) |
| None of the above | 128 (21.7%) |
| Preoperative use of antithrombotic medication |  |
| No | 310 (52.6%) |
| Antiplatelet | 131 (22.2%) |
| Anticoagulation | 143 (24.3%) |
| Both | 5 (0.8%) |
| History of head trauma |  |
| Yes | 442 (75.0%) |
| No | 56 (9.5%) |
| Unknown | 91 (15.4%) |
| GCS at admission |  |
| 15 | 465 (78.9%) |
| 14 | 88 (14.9%) |
| 9–13 | 36 (6.1%) |
| mRS score at admission |  |
| 1–3 | 388 (65.9%) |
| 4–5 | 201 (34.1%) |
| Hematoma laterality^c^ |  |
| Unilateral | 439 (74.5%) |
| Bilateral | 149 (25.3%) |
| Midline shift, median (IQR), mm | 7 (3–7) |
| Hematoma width,^d^ median (IQR), mm | 24 (19–32) |
| Randomized group |  |
| Irrigation | 294 (49.9%) |
| No irrigation | 295 (50.1%) |
| Reoperation within 6 months | 91 (15.4%) |
| Thromboembolic complication | 33 (5.6%) |
| Hemorrhagic complication | 18 (3.1%) |
| ^a^One patient can have several comorbidities. | |
| ^b^Medication used within 12 months before admission. | |
| ^c^Patient can have a hematoma not operated on. | |
| ^d^Sum of left and right hematoma widths for bilateral hematomas. | |
| Abbreviations: IQR, interquartile range; GCS, Glasgow Coma Scale; mRS, modified Rankin Scale; mm, millimeter. | |

## Supplementary Figures

**eFigure 1.** Survival by preoperative comorbidity

### eFigure 2A. Number of deaths for different causes of death


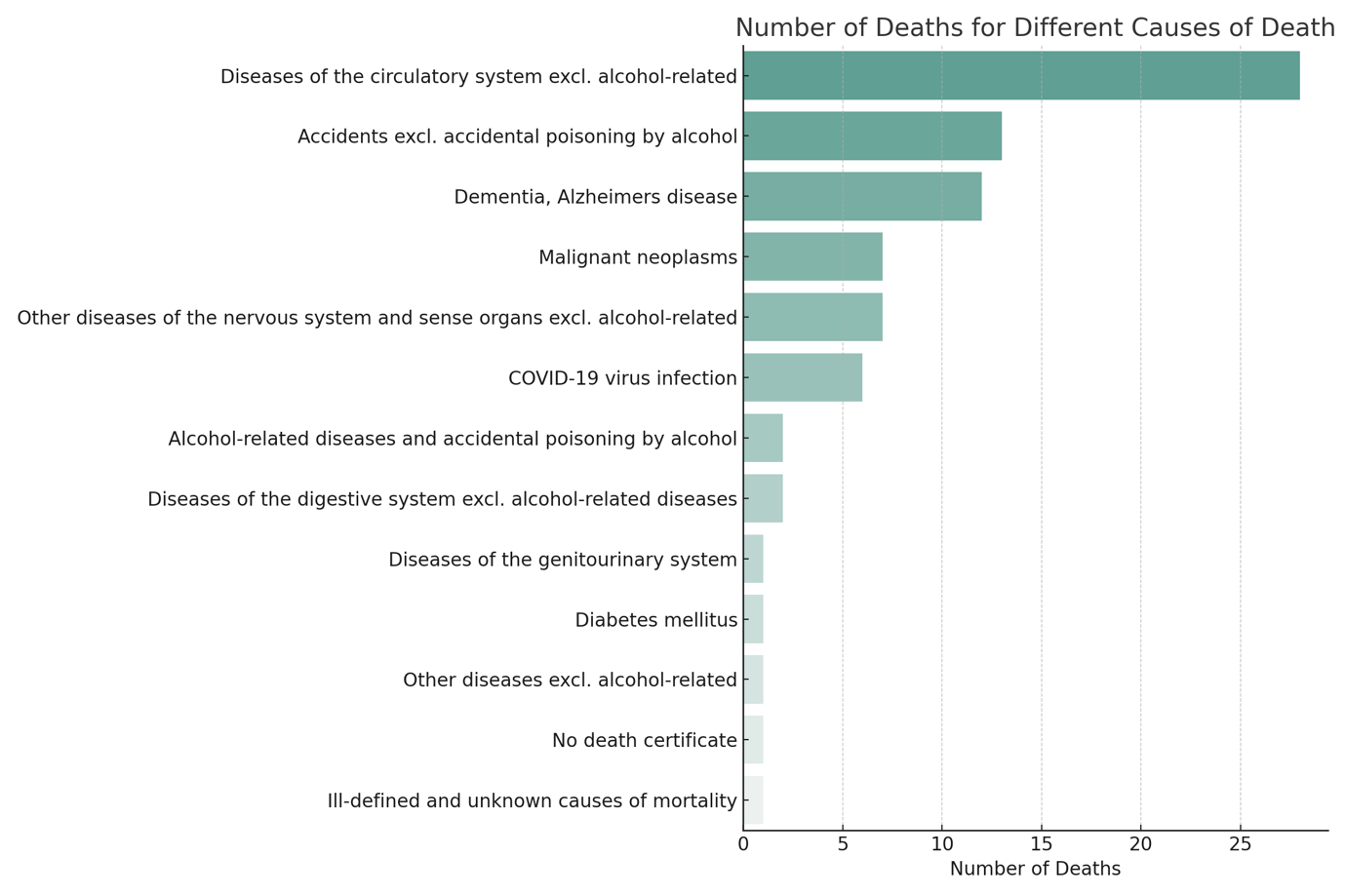


### eFigure 2B. Time to death from surgery for different causes of death


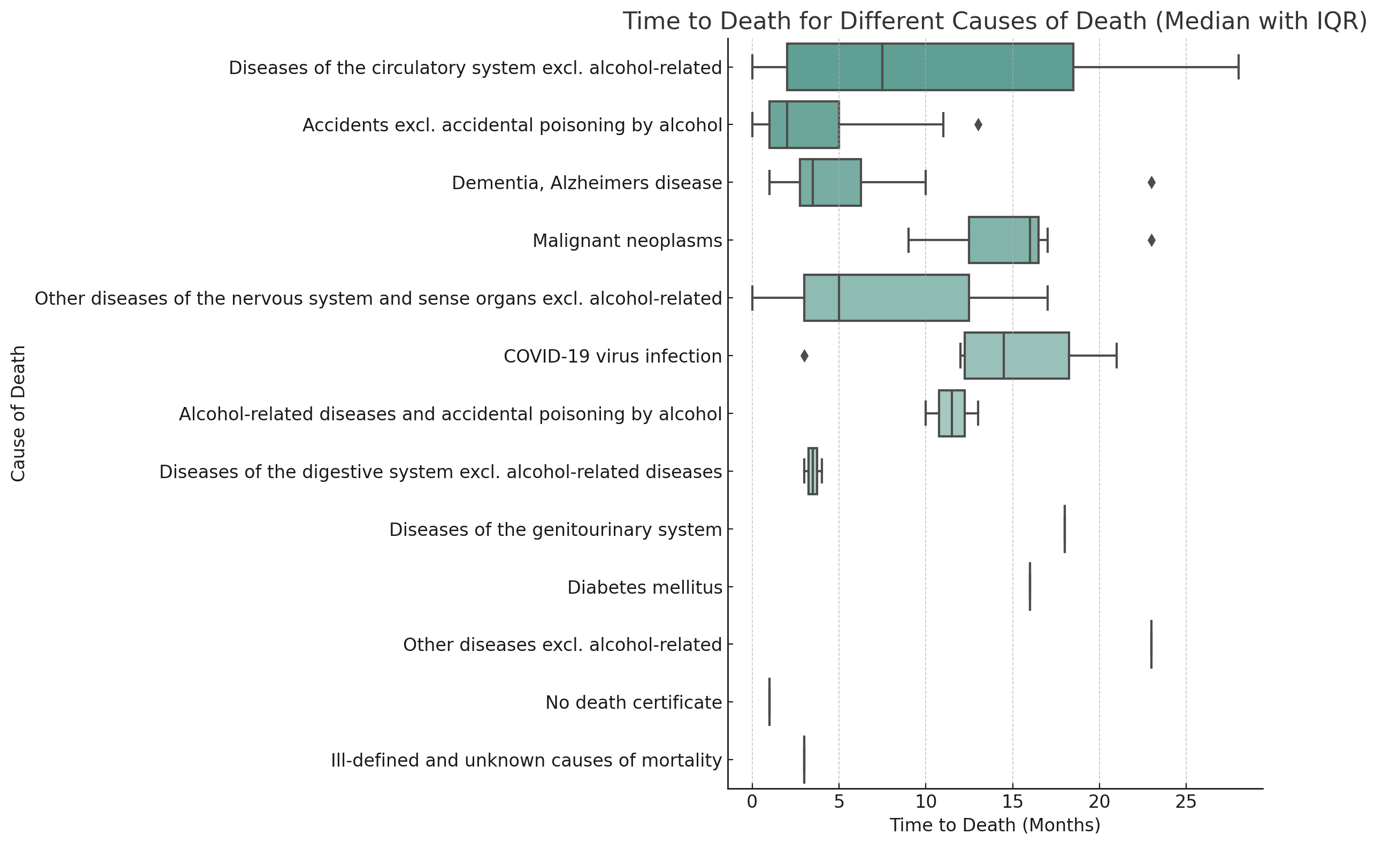


## Complete list of FINISH investigators *(to be indexed on Pubmed*)*:*

| **First and middle names or initials** | **Surnames** |
| --- | --- |
| Abdirisak | Ahmed |
| Tarmo | Areda |
| Jiri Jr | Bartek |
| Tomasz | Czuba |
| Nils | Danner |
| Antti-Pekka | Elomaa |
| Janek | Frantzén |
| Ilkka | Haapala |
| Joonas | Haapasalo |
| Juuso | Heikkilä |
| Minttu | Hellman |
| Henna | Henttonen |
| Nora | Huuska |
| Teppo LN | Järvinen |
| Henna-Kaisa | Jyrkkänen |
| Aku | Kaipainen |
| Olli-Pekka | Kämäräinen |
| Hanna | Kämppi |
| Milla | Kelahaara |
| Riku | Kivisaari |
| Nikolai | Klimko |
| Oula A | Knuutinen |
| Timo | Koivisto |
| Tommi | Korhonen |
| Janne | Koskimäki |
| Anselmi | Kovalainen |
| Xenia | Kuparinen |
| Dan | Laukka |
| Martin | Lehecka |
| Kai | Lehtimäki |
| Ville | Leinonen |
| Kimmo | Lönnrot |
| Antti | Luikku |
| Teemu | Luostarinen |
| Teemu | Luoto |
| Janne | Luotonen |
| Lauriina | Lustig-Tammi |
| Henna-Riikka | Maanpää |
| Jenni | Määttä |
| Timo | Möttönen |
| Eliisa | Netti |
| Laura | Nevaharju-Sarantis |
| Mika | Niemelä |
| Tero | Niskakangas |
| Mette | Nissinen |
| Ville | Nurminen |
| Minna | Oinas |
| Teemu | Ollonen |
| Anna | Östberg |
| Elias | Oulasvirta |
| Krista | Pantzar |
| Katri | Piilonen |
| Anni | Pohjola |
| Markus | Polvivaara |
| Jussi P | Posti |
| Rahul | Raj |
| Linnea | Rajala |
| Jonas | Ranstam |
| Minna | Rauhala |
| Behnam | Rezai-Jahromi |
| Miika | Roiha |
| Ilkka | Saarenpää |
| Antti | Sajanti |
| Henrikki | Salmi |
| Jarno | Satopää |
| Christoph | Schwartz |
| Niina | Shemeikka |
| Pia | Sorto |
| Simo | Taimela |
| Sami | Tetri |
| Tuomo | Thesleff |
| Pihla | Tommiska |
| Maarit | Tuomisto |
| Nuutti | Vartiainen |
| Ville | Vasankari |
| Jyri | Virta |
| Mikko | Visuri |
| Paula | Walle |
| Frederick A | Zeiler |
